# Applying spatio-temporal models to assess variations across health care areas and regions: Lessons from the decentralized Spanish National Health System

**DOI:** 10.1371/journal.pone.0170480

**Published:** 2017-02-06

**Authors:** Julián Librero, Berta Ibañez, Natalia Martínez-Lizaga, Salvador Peiró, Enrique Bernal-Delgado, on behalf of the Spanish Atlas of Medical Practice Variation Research Group

**Affiliations:** 1 Navarrabiomed—Fundación Miguel Servet, Pamplona, Spain; 2 Red de Investigación en Servicios de Salud en Enfermedades Crónicas (REDISSEC), Bilbao, Spain; 3 Instituto Aragonés de Ciencias de la Salud, IIS Aragón, Zaragoza, Spain; 4 Centro Superior de Investigación en Salud Pública (CSISP-FISABIO), Valencia, Spain; Liverpool School of Tropical Medicine, UNITED KINGDOM

## Abstract

**Objective:**

To illustrate the ability of hierarchical Bayesian spatio-temporal models in capturing different geo-temporal structures in order to explain hospital risk variations using three different conditions: Percutaneous Coronary Intervention (PCI), Colectomy in Colorectal Cancer (CCC) and Chronic Obstructive Pulmonary Disease (COPD).

**Research design:**

This is an observational population-based spatio-temporal study, from 2002 to 2013, with a two-level geographical structure, Autonomous Communities (AC) and Health Care Areas (HA).

**Setting:**

The Spanish National Health System, a quasi-federal structure with 17 regional governments (AC) with full responsibility in planning and financing, and 203 HA providing hospital and primary care to a defined population.

**Methods:**

A poisson-log normal mixed model in the Bayesian framework was fitted using the INLA efficient estimation procedure.

**Measures:**

The spatio-temporal hospitalization relative risks, the evolution of their variation, and the relative contribution (fraction of variation) of each of the model components (AC, HA, year and interaction AC-year).

**Results:**

Following PCI-CCC-CODP order, the three conditions show differences in the initial hospitalization rates (from 4 to 21 per 10,000 person-years) and in their trends (upward, inverted V shape, downward). Most of the risk variation is captured by phenomena occurring at the HA level (fraction variance: 51.6, 54.7 and 56.9%). At AC level, the risk of PCI hospitalization follow a heterogeneous ascending dynamic (interaction AC-year: 17.7%), whereas in COPD the AC role is more homogenous and important (37%).

**Conclusions:**

In a system where the decisions loci are differentiated, the spatio-temporal modeling allows to assess the dynamic relative role of different levels of decision and their influence on health outcomes.

## Introduction

There is growing need for the assessment of health systems performance as a mean to improve their effectiveness, resilience and sustainability. Typically, the focus of performance analyses is put on country-average (at the most regional analyses) where measures talk about care provision, mainly, hospital care [1]. However, the study of variations in performance attributable to different decision levels is getting momentum [2–4] overtaken the classical country-average oriented approach present in international reporting and turning the emphasis into the analysis of variations at sub-country (e.g. regions) and sub-regional levels (e.g., health care areas).

Classical methods for the study of geographic variations in health care performance assume: 1) independence between neighbouring areas (i.e., performance in area x does not depend on performance in area y); 2) independence across areas within a region (i.e., no existence of regional contextual phenomena affecting areas, differential across regions); 3) time-independence (i.e., performance in an area x in t, is independent of performance in t-1); and, 4) over the years, performance evolution is deemed homogeneous across areas or regions (i.e., time-dependent phenomena spread homogeneously). However, looking at how actually systems are organized and perform, it is hard to adopt those assumptions when estimating health system performance, particularly in decentralized systems. Such an assumption would imply to accept that, for example: a) differences in the standardized rate of percutaneous coronary interventions across areas are exclusively attributable to phenomena confined to the area, (i.e., omitting that all the areas might be served by the same catheterization laboratory); b), the epidemiology of coronary disease is not shared by neighbour areas; c) the adoption of new tech or the learning curves does not exist; or d) that there are no healthcare policies affecting similarly all the areas within a region [5–7].

The Spanish National Health System (SNHS) is one of these quasi-federal decentralized systems [8,9], where 17 regional governments have full responsibility on policy-making, planning and financing at regional level, and in turn, each region is organized in health care areas (HA), the locus for hospital *and primary care* provision. Hence, regions build a second spatial grid, and it is of interest to assess their influence on the level and the dynamic of some health outcomes such as hospitalization risks.

Using three different conditions, this paper shows the application of well known disease-mapping methodology to thoroughly describe spatio-temporal hospitalization risks, overcoming some of the aforementioned hindrances, while taking into consideration the spatiotemporal structure of the phenomena under study and different sources of variation: regional level (Autonomous Community, AC), health care areas (HA) level, and time phenomena that homogeneously occurs across regions (average behaviour) or unevenly across regions (interaction), over a period of time.

## Methods

### Design

This is a population-based spatio-temporal study with a two-level geographical structure; the first composed of the HA and the second of the AC.

### Setting

The Spanish National Health System (SNHS) is an almost universal system with a decentralized structure of 17 regional National Health Services administered by the 17 Autonomous Governments of the Spanish regions [8,9]. Regional services manages an extensive network of hospitals (about 75% of acute hospital beds in Spain), and specialized outpatient and primary healthcare centres. In 2013 the SNHS was organized into 203 HAs, geographical territories –50% of them between 80.000 and 263.000; median 159,000 inhabitants–served by one hospital that provides specialized inpatient and outpatient care to the residents in its area. The strong hierarchy (HAs are nested into AC) and the neat differentiation of roles between both geographic levels provide an empirical context wherein the properties of this kind of analysis can be safely assessed.

### Main outcome measures

Main outcome measures are the spatio-temporal hospitalization relative risks, the evolution of the variation, and the relative contribution (*i*.*e*., fraction of variation) of each of the model parameters in the explanation of the variation in hospitalization risks.

For the purpose of this study, we evaluate the hospitalization risks for three selected conditions (Percutaneous Coronary Intervention (PCI), Colectomy in Colorectal Cancer (CCC) and Chronic Obstructive Pulmonary Disease (COPD)), in patients aged 20 and older, discharged from 2002 to 2013. The three selected conditions illustrate how spatio-temporal modeling may capture different geo-temporal structures in the explanation of hospitalization risk variation, as the relative importance of the spatial terms (both, HAs and AC), the average-trend, and the spatio-temporal interaction (see next section).

### Statistical analysis

To describe the data, age and sex-standardized rates per 10,000 person-years were obtained for each *i-th* HA and *t-th* year by each condition. Standardized Hospitalization ratios were also estimated for each condition using the ratio of observed (o_it_) to expected (e_it_) cases, were expected cases per HA and year were estimated by applying the rate for the whole region and period to the population at risk of each HA. Variability among HA was quantified using both the extremal quotient excluding areas outside the percentiles 5 and 95 and the Systematic Coefficient of Variation [10].

### Spatio-temporal analysis

Several methods to model spatio-temporal data structures have been developed in the last two decades within the disease mapping framework [11,12], and nowadays are being extensively used to describe the temporal evolution of geographical patterns of mortality risk or rates [13,14]. Whereas the most widely-used approach has been to fit spatio-temporal models formulated within a hierarchical Bayesian framework under the fully Bayes approach based on Markov Chain Monte Carlo (MCMC) methods [15,16], nowadays a novel technique called integrated nested Laplace approximations (INLA) has become an alternative estimation procedure, which overcome some of the limitations of the MCMC methods, such as the computation burden needed when tackling with high-dimension datasets [13,17,18].

In this work a spatio-temporal model that accounts both for the spatial nested structured of dependence of the HA within the ACs and for the temporal dependence was fitted within the INLA framework. The model assumes that, conditional on the underlying relative risk *r*_*it*_, the number of counts in each area and time period, o_it_, follows a Poisson distribution, namely,

$$o_{it}∼Poisson\;(\mu_{it}=e_{it}r_{it});\;log{\;\mu }_{it}=\log\;e_{it}+\log\;r_{it}$$

The log-risk is modeled taking into account the need of distinguishing between space (both in the AC and HA levels) and time components, and including interaction in space and time to allow each AC to have its own trajectory. More precisely, we assumed that

$$\log\;r_{it}=\beta +u_{i(j)}+v_{j}+\gamma_{t}+\delta_{jt},$$

where *β* is an overall risk level, *u*_*i*(*j*)_ represents the spatial level for the i-th HA area which belongs to the j-th AC region, *v*_*j*_ represents the spatial level for the j-th AC region, *γ*_*t*_ denotes temporal effects, and *δ*_*jt*_ are space-time interaction effects. Gaussian exchangeable distributions were assumed for the spatial random effects ***u*** and ***v***, whereas for the temporal effect ***γ***, a dynamic neighboring structure using a random walk of order 1, was used. That means that in space, each HA may have its own risk, but all HA within an AC region share a common spatial effect, whereas in time, each year has two neighbors, the previous point and the following one, except for the first and last year, which only depends on one. For the interaction term *δ*, we assume that the effects ***v*** and ***γ*** interact assuming an exchangeable distribution, *δ*_*jt*_ ∼ *N*(0,*τ*_*δ*_), with *τ*_*δ*_ the precision parameter of the random effect. Minimally informative priors were specified on the log of the precision parameters, log(*τ*) ∼*logGamma*(1,0.0005). Calibration of the model was performed using the probability integral transform histogram [19]. The evolution of the variation was assessed using the estimation of risks variance in each year. Lastly, the relative contribution of each model component was assessed throughout the estimation of the fractions of variance. Sensitivity analyses were also conducted to determine the effect of observations with singular behavior on the different fractions of variation.

In summary, the model we fitted allows to assess the heterogeneity in the regional dynamic of hospitalization risks. Other specifications could be adopted to assess related research questions. For comparative purposes, we also fitted an alternative model which incorporates the neighborhood structure in the estimation procedure. We visually compared their latent components and assessed the agreement between the results of both models using the Intraclass Correlation Coeficient (ICC) for the estimated relative risks. Details of the model and of the comparison study are given in S2 File).

Codes of the statistical analyses are available at https://dx.doi.org/10.6084/m9.figshare.4585042. All analysis and graphs were performed in R, version 3.2.3, and models were implemented in this program via the library R-INLA, available on the website http://www.r-inla.org.

### Sources of data

We used data from the Atlas of Variations in Medical Practice in the SNHS (namely, “AtlasVPM”), a research project designed to inform Spanish decision-makers on unwarranted variations in health care performance (see www://atlasvpm.org). The project build upon administrative data on hospital admissions, which provides clinical and socio-demographic information on all hospital discharges in the SNHS, including diagnoses and procedures, coded according to the International Classification of Diseases 9^th^ revision Clinical Modification. Post-codes were used to allocate every admission to the Healthcare Area where the patient lives. Population data were extracted from the Spanish National Institute of Statistics’ Municipal Register of Inhabitants for each year. Datasets available at http://www.atlasvpm.org/articulos

### Ethical aspects

This study, observational in design, uses retrospective anonymized non-identifiable and non-traceable data, and was conducted in accordance with the amended Helsinki Declaration, the International Guidelines for Ethical Review of Epidemiological Studies, and Spanish laws on data protection and patients’ rights. The study was reviewed and approved by the Clinical Research Ethics Committee of Aragon (CEICA), who waived the need for written informed consent from the participants.

## Results

The evolution of the population risk of hospitalization for each condition is shown in Table 1. For CCC, the age and sex-standardized rates per 10,000 person-years keep around 4 along the whole period, whereas for COPD they declined from 21 to 15 and for PCI they rose from 8 to 13. Classical variation statistics show low and constant geographical variability in CCC, and high initial geographical variability that diminished in PCI and CODP.

**Table 1:** Number of cases, admission rates per 10,000 person-years and variation statistics for each condition and year.

	2002	2003	2004	2005	2006	2007	2008	2009	2010	2011	2012	2013
**PCI**												
cases	27,566	31,919	35,837	39,621	42,696	45,317	44,656	48,184	51,695	49,935	51,033	53,372
rates	7.70	8.74	9.68	10.48	11.12	11.67	11.31	12.10	12.94	12.47	12.74	13.37
EQ_5-95_	6.44	4.60	4.45	4.62	5.13	4.70	5.83	3.86	4.19	4.47	3.58	3.27
SCV	0.20	0.18	0.18	0.19	0.19	0.19	0.21	0.17	0.14	0.12	0.10	0.08
**CCC**												
cases	14,990	16,055	16,652	17,258	17,773	18,673	19,217	20,311	17,153	17,347	16,797	16,973
rates	4.19	4.39	4.50	4.57	4.63	4.81	4.87	5.10	4.29	4.33	4.19	4.25
EQ_5-95_	2.76	2.36	2.26	2.18	2.37	2.02	2.02	2.00	2.09	2.12	2.42	2.32
SCV	0.06	0.04	0.04	0.02	0.07	0.03	0.03	0.03	0.03	0.03	0.04	0.04
**COPD**												
cases	75,084	77,935	72,333	80,081	68,667	77,255	72,698	71,012	64,625	65,373	65,005	61,393
rates	20.98	21.33	19.54	21.19	17.88	19.89	18.41	17.84	16.17	16.32	16.22	15.38
EQ_5-95_	6.86	4.97	5.57	4.96	4.67	4.75	4.53	5.10	4.71	4.49	4.22	4.61
SCV	0.29	0.24	0.23	0.23	0.23	0.23	0.19	0.19	0.20	0.16	0.18	0.16

**PCI**: Percutaneous Coronary Inervention; **CCC**: Colectomy in colorectal cancer; **COPD**: Chronic Obstructive Pulmonary Disease; **EQ**_**5-95**_: Extremal Quotient; **SCV:** Systematic Coefficient of Variation.

**Fig 1:**
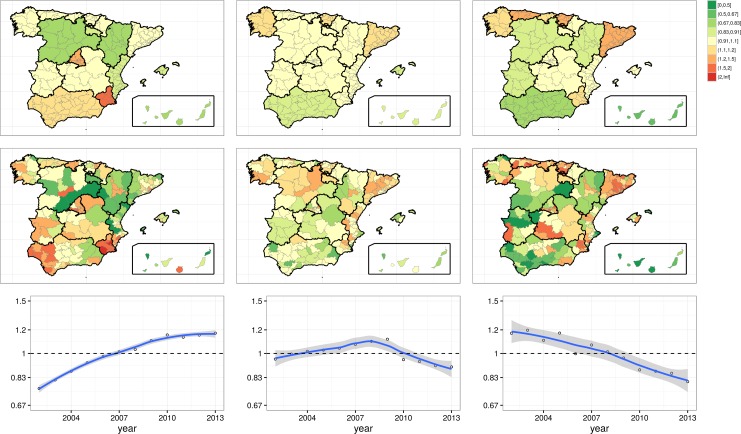
Hospitalization risk maps of the spatial region effect -upper row, exp(*v*_*j*_)- and of the global spatial component -middle row, exp(*v*_*j*_ + *u*_*i*(*j*)_)- for Percutaneous Coronary Intervention (PCI; left), Colectomy in Colorectal Cancer (CCC; middle) and Chronic Obstructive Pulmonary Disease (COPD, right). At the bottom row, the average temporal trend exp (*γ*_*t*_) (2002–2013).

Fig 1 shows the spatial component in the variation of hospitalization risks and the average time evolution of risks: AC (*v*_*j*_), joint HA plus AC (*u*_*i*(*j*)_ + *v*_*j*_) level, and average time (*γ*_*t*_), for each condition. In the upper row, unlike PCI and COPD, the homogeneity of risk ratios in CCC at AC level is noticeable, with only 6 out of 17 ACs with risk ratios slightly over or below 1. In the middle row, hospitalization risks show rather scattered patterns in PCI and COPD, less obvious in CCC. The joint observation suggests a higher contribution of HA fraction in the explanation of the three conditions, and a lower contribution of the AC fraction in CCC. With regard to time component, PCI and COPD exhibited opposite quasi-lineal trends; in turn, CCC shows an inverted-V shape with a time trend change in 2010. In average, the magnitude of variation in risks in the time component is lower (maximum range of risk values for exp(*γ*_*t*_) between 0.76 to 1.20) than that observed in the spatial components (maximum range between 0.25 to 2.44).

**Fig 2:**
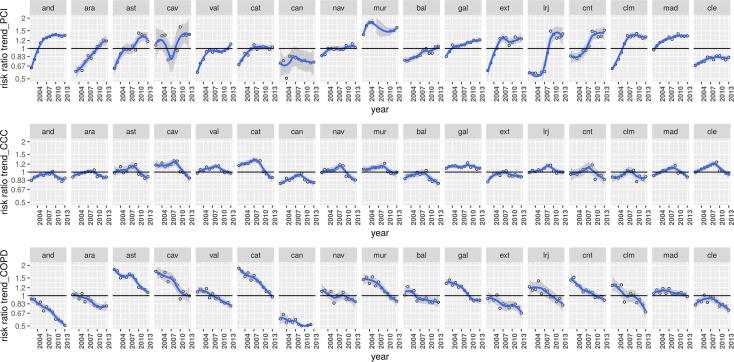
Temporal trends for hospitalization risks—exp(*v*_*j*_ + *γ*_*t*_ + *δ*_*jt*_)—in each of the 17 regions along 2002–2013 for Percutaneous Coronary Intervention (PCI; upper row), Colectomy in Colorectal Cancer (CCC; middle row) and Chronic Obstructive Pulmonary Disease (COPD; lower row).

In Fig 2 (exp(*v*_*j*_ + *γ*_*t*_ + *δ*_*jt*_)), a different evolution across ACs is observed. This finding points out to a heterogeneous regional behaviour of hospitalization risks beyond the average. While in CCC and COPD the trend shape is fairly homogenous in all ACs (COPD decreasing and CCC inverted-V), in the case of PCI the trend shape is observed to vary across ACs.

**Fig 3:**
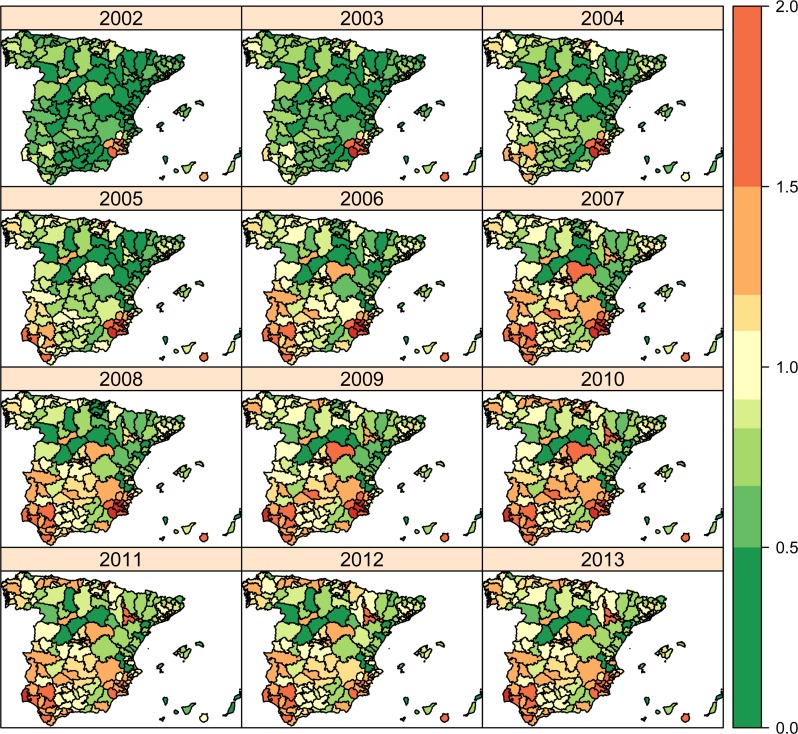
Space-time relative risk estimates (exp(*β* + *u*_*i*(*j*)_ + *v*_*j*_ + *γ*_*t*_ + *δ*_*jt*_)) for Percutaneous Coronary Intervention (PCI).

**Fig 4:**
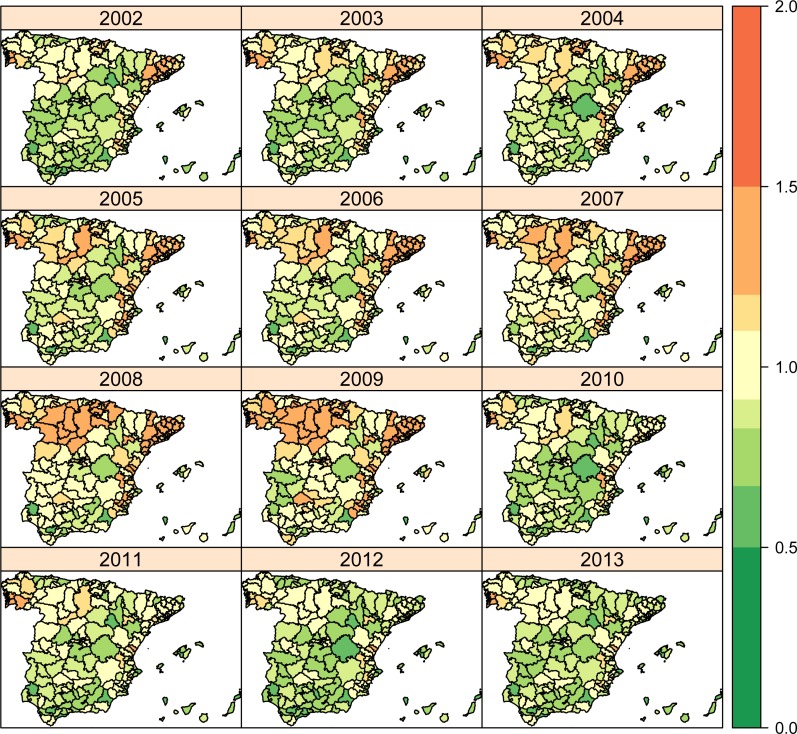
Space-time relative risk estimates (exp(*β* + *u*_*i*(*j*)_ + *v*_*j*_ + *γ*_*t*_ + *δ*_*jt*_)) for Colectomy in Colorectal Cancer (CCC).

**Fig 5:**
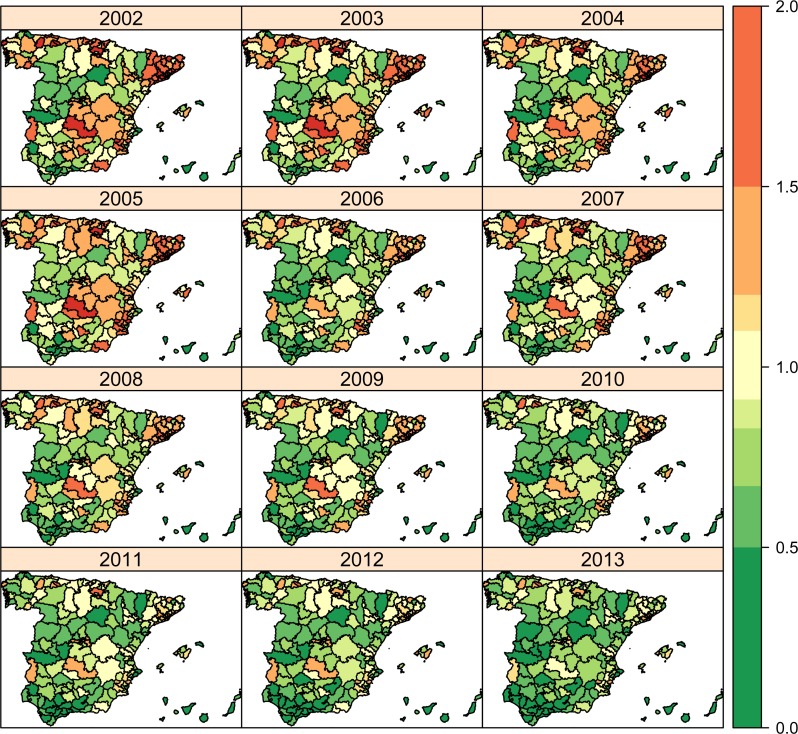
Chronic Obstructive Pulmonary Disease (COPD) admissions along 2002–2013.

In Figs 3 to 5, global risk patterns including spatial (HA and AC) and temporal parameters (average trend and interaction term) are shown (exp(*β* + *u*_*i*(*j*)_ + *v*_*j*_ + *γ*_*t*_ + *δ*_*jt*_)) for each condition. For PCI (Fig 3), although green colours are predominant at the beginning of the period as a reflection of low and relatively homogeneous hospitalization risks, the pattern scatters at the middle of the period and it holds until the end of it. In accordance, variability in spatial risks (yearly risk variance) slightly grows up from the 0.068 in 2002 to the 0.113 in 2013 (data not shown). In the case of CCC (Fig 4), the inverted U-shape of the average trend is observed, with light green colours at the two extremes of the series, while the hospitalization risks appear less scattered than PCI, and an AC-wise pattern is observed in the central years of the series, pointing out to a more prominent effect of ACs at the moment. Variability for CCC risks practically holds between 2002 to 2008 (0.032 to 0.034, respectively), to sharply decrease to 0.018 by 2013. In COPD (Fig 5), in turn, a stronger AC-wise pattern with notable heterogeneity across HAs is observed the first years, diluting over time to converge to a green coloured map that reflects the decreasing average trend on hospitalization risks. The evolution of the risk variation for COPD is, therefore, drastically reduced along time, from 0.326 in 2002 to 0.098 in 2013.

Table 2 shows the variance decomposition across the three conditions. Consistent with the observations in Figs 1 to 5, most of the variation is captured by phenomena occurring at HA level (51.6% in PCI, 54.7% in colectomy, and 56.9% in COPD). Interestingly, the distribution of variance varies significantly across conditions. In PCI the spatio-temporal interaction (i.e., the different behaviour of ACs over time) explained a 17.7% of the variation, almost two-fold the explanation of CCC variance and almost 6 times that one for COPD, consistent with what it is observed in Fig 2. In COPD, the spatial effect at AC level implied a higher fraction of variation (37%). In CCC, the global temporal trend (i.e., average behaviour) showed a fairly higher impact in the explanation of variation, in accordance to its inverted-V shape, widespread observed across regions.

**Table 2:** Variance decomposition according to the model fitted per each condition (left hand side) and sensibility analysis excluding regions with singular behavior.

	With all regions			Excluding singular values	
	PCI	CCC	COPD	PCI_(Cav, Mur)_	COPD_(Can)_
**Spatial HA **	51.6%	54.7%	56.9%	70.1%	67.3%
**Spatial AC**	28.6%	26.6%	37.0%	12.4%	25.3%
**Temporal **	2.1%	9.1%	3.0%	2.8%	3.7%
**Spatio-temp**	17.7%	9. 6%	3.1%	14.7%	3.7%

**PCI**: Percutaneous Coronary Inervention; **CCC**: Colectomy in colorectal cancer; **COPD**: Chronic Obstructive Pulmonary Disease; **PCI**_**(Cav, Mur**_**):** PCI excluding the Comunidad Autónoma Vasca and Murcia Data, **COPD**_**(Can)**_: COPD excluding the Canarias Data.

Once discarded the effect of ACs with singular behaviour (in Fig 2, Cav and Mur in PCI, and Can in COPD), most of the variation of PCI was shown at HA level (70.1%) holding a significant 14.7% when it comes to the spatio-temporal interaction. In the case of COPD, figures for the spatial fractions also changed, increasing the attribution to HA (from a 56.9% to a 67.3%) and reducing the AC attribution, from a 37% to a 25.3%.

Results of the model including the neighbourhood structure in the estimation procedure are given in S2 File, and show a high degree of agreement with those derived from the model presented here.

## Discussion

This empirical exercise, meant to illustrate the use of disease mapping techniques in the assessment of spatio-temporal hospitalization risks in a decentralized and hierarchically organized health care system, has yielded two lessons for the application of this technique: a) the methodology is flexible and allows to model and elicit different sources of variation; b) the methodology allows eliciting differences across conditions; and, as consequence, in a system where the decisions *loci* are differentiated, it allows to analyse the different levels of decision.

### The methodology is flexible and allows modelling different sources of variation

First, the wide range of model specification structures offered within the spatio-temporal framework allows us to adapt to practically any research question we may formulate and any casuistic we may encounter in practice. It may include the presence of a hierarchical geographical structure, or other spatial structures of dependence such as vicinity. It also allows for different temporal structures, from linear or quadratic parametric formulas till more relaxed forms such as autoregressive or independent, as the used in this application. And furthermore, it allows specifying different trends for different geographical areas, by means of the interaction terms that, in turn, can have different specifications [19]. In our case of study, the focus was to assess the influence of regional level on the dynamic of the hospitalization risk pattern, attending to the nested structure of HAs within ACs, and the chosen specification has allowed us to easily capture this latent phenomenon. As we have shown in the S2 File, it is possible to adopt other widely used specifications that account for the spatial neighborhood structure, whose results, in general terms, highly agree with those derived from our model, and that can be used to assess related research questions.

Second, the methodology is able to capture the presence of geographical areas (e.g., ACs) with trajectories that substantially differ from the global dynamic structure, as it is sensible to their presence, as can be seen when looking at the impact of including them in the estimation of fractions of variance (see comments to Fig 2 and Table 2).

### The methodology allows eliciting differences across conditions and decision levels

The selection of three conditions was meant to allow the different parameters in the model to play a differential role. While the HA-spatial term is expected to capture most of the variation in any of the conditions (fairly above 50%, even higher, excluding outlier ACs), the spatio-temporal interaction term is expected to behave differently across conditions.

Our results show that this methodology has been sensitive to the different underlying phenomena for these three conditions in the period of study, 2002 to 2013. As a matter of examples: 1) as expected, a higher proportion of variation attributable to the interaction term has been observed in PCI, due to the uneven adoption of innovations (i.e., primary PCI) in the last decade; 2) a smaller proportion of variation attributed the AC (spatial plus interaction) has been observed in COPD, given that decisions on how to manage these patients are mainly taken at HA level and there have not been abrupt innovations on patients’ treatment along the period of study; and 3) the average-temporal trend have had a predominant participation in the explanation of CCC variation given the sharp adoption of endoscopic treatment in the late 2000’s, as a consequence of colorectal cancer screening programs.

### Caveats for its application and interpretation

In spite of the properties of this methodology, the statistical research devoted to identify those geographic areas with discrepant tendencies is still limited and not easily applicable to all practical contexts. Nevertheless, there are some promising approaches on cluster detection published in recent years [20,21].

On the other hand, when interpreting fractions of variation, it should also be considered that this may be influenced by the number of units of analysis (in our case 203 HA *vs*. 17 AC and 12 years) and the number of parameters, and further, it is relative measure that does not allow comparisons in absolute terms. So, the relatively lower effect of the average-temporal trend observed in this paper, rather than a true low impact, might reflect that the estimated temporal effect *γ*_*t*_ barely varies as compared to the geographical variation in *u*_*i*(*j*)_.

And finally, results are contextual-dependent. To assess generalizability of our results (in terms, for instance, of the relative effect of each parameter in the modelling of the spatio-temporal evolution of these three conditions), replications with local data should be conducted.

### Conclusion

In a system where the decisions loci are differentiated, the Bayesian spatio-temporal modeling enables the assessment of the role and dynamics of different levels of decision and their influence on health care events, such as hospital admissions.

## Supporting information

S1 File Spanish Atlas of Medical Practice Variation Research Group. (DOCX)

S2 File Comparison study between the adopted model and other possible specification accounting for the neighborhood structure. (DOCX)
