# Supplementary material for: Applying spatio-temporal models to assess variations across health care areas and regions: Lessons from the decentralized Spanish National Health System
Source: PLoS One. 2017 Feb 6;12(2):e0170480. doi: 10.1371/journal.pone.0170480 (PMC5293276; doi:10.1371/journal.pone.0170480)
Supplement: S1 File — (DOCX) [file pone.0170480.s001.docx]

**The Spanish Atlas of Medical Practice Variation Research Group** is formed by ***ANDALUSIA***: Díaz Martínez A (Hospital Virgen del Rocio, Sevilla), Goicoechea Salazar JA (Servicio Andaluz de Salud, Sevilla), Rivas Ruiz F, Jiménez Puente A (Hospital Costa del Sol, Marbella), Rodríguez del Águila MM (Hospital Virgen de las Nieves, Granada), Molina T, Baños E (Agencia de Evaluación de Tecnologías Sanitarias de Andalucía, Sevilla); ***ARAGON***: Angulo E, Bernal Delgado E, Comendeiro Maaløe M, Estupiñán Romero FR, García Armesto S, Launa R, Martínez Lizaga N, Ridao M, Seral Rodríguez M (Instituto Aragonés de Ciencias de la Salud-Instituto de Investigación Sanitaria Aragón, Zaragoza), Abad Diez JM, Arribas Monzón F, Beltrán Peribáñez J, Pradas Arnal F (Departamento de Sanidad, Bienestar Social y Familia, Gobierno de Aragón, Zaragoza) ;***ASTURIAS***: Caicoya M, Suárez F (Consejería de Sanidad. Principado de Asturias, Oviedo); ***CANARY ISLANDS***: Sánchez Janáriz H, Alonso Bilbao Jl, Fiuza Pérez D, (Servicio Canario de la Salud, Las Palmas de Gran Canaria); ***CANTABRIA***: Romero G (Consejería de Sanidad, Santander). ***CATALONIA***: Marinelli M (Agència de Qualitat i Avaluació Sanitàries de Catalunya, AQuAS, Barcelona), Oliva G (Departament de Salut, Barcelona), Ortún Rubio V (Universitat Pompeu Fabra, Barcelona); Salas T, Vela E (CatSalut- Servei Català de la Salut, Barcelona); ***CASTILLA-LEON***: Sacristán Salgado A, García Crespo J (Dirección General de Desarrollo Sanitario, Valladolid), Melgosa Arcos A, Sangrador Arenas L (Dirección General de Planificación, Calidad, Ordenación y Formación, Valladolid); ***CASTILLA LA MANCHA***: García Sánchez MA (Consejería de Sanidad y AS de Castilla- La Mancha, Toledo); López Reneo R (Servicio Salud Castilla-La Mancha, SESCAM, Toledo), ***GALICIA***: Atienza Merino G, Carballeira Roca C, Queiro T (Conselleria de Sanidade de la Xunta de Galicia, Santiago), Castro Villares M (Servicio Galego de Saúde, Santiago); ***ESTREMADURA***: Anes del Amo Y (Consejería de Salud y Política Social, Extremadura, Mérida), Montes Salas G (Escuela de Estudios de Ciencias de la Salud, Badajoz); ***BALEARIC ISLANDS***: Castaño Riera EJ, Santos Terrón MJ, (Consejería de Salud, Palma); Zaforteza Dezcallar M (Servicio de Salud de las Illes Balears, Palma), Ferrer Riera J, Martín Martín MV (Hospital Son Llàtzer, Palma); ***RIOJA***: Cestafé A (Consejería de Salud, Logroño); ***MADRID***: Bienzobas López C, Gómez Lázaro R (Dirección General de Sistemas de Información Sanitaria, SERMAS, Madrid); ***MURCIA***: Palomar Rodríguez J, Hernando Arizaleta L (Consejería de Sanidad de la Región de Murcia, Murcia); ***NAVARRE***: Álvarez Arruti N, Montes García Y, Rodrigo Rincón I (Departamento de Salud de Navarra- Osasunbidea, Pamplona), Ibáñez Beroiz B (Centro de Investigación Biomédica-Navarra, Pamplona); ***BASQUE COUNTRY***: Aizpuru F, Latorre García PM, Latorre A, Pérez de Arriba J (Grupo de investigación del País Vasco, Osakidetza-SVS, Gazteitz), Errezola M (Departamento de Sanidad del Gobierno Vasco, Gazteitz),Millán E (Osakidetza-SVS, Gazteitz); ***VALENCIA COMMUNITY***: Baixauli-Pérez C, Librero J, Peiró S, Rodríguez-Bernal CL,Sanfelix-Gimeno G (Fundación para el Fomento de la Investigación Sanitaria y Biomédica de la Comunidad Valenciana, FISABIO, Valencia), Meneu R, Sotoca R (Fundación Instituto de Investigación en Servicios de Salud, fIISS, Valencia),Calabuig J (Conselleria de Sanitat, Generalitat Valenciana, Valencia).
