# Supplementary material for: Applying spatio-temporal models to assess variations across health care areas and regions: Lessons from the decentralized Spanish National Health System
Source: PLoS One. 2017 Feb 6;12(2):e0170480. doi: 10.1371/journal.pone.0170480 (PMC5293276; doi:10.1371/journal.pone.0170480)
Supplement: S2 File — (DOCX) [file pone.0170480.s002.docx]

**Applying Spatio-Temporal Models to Assess Variations across Health Care Areas and Regions: Lessons from the Decentralized Spanish National Health System**

**S2: Comparison study between the adopted model (model 1) and other possible specification accounting for the neighborhood structure (model 2)**

***Comparison of the characteristics of Model 1 and Model 2***

In table S1 we specify the model characteristics of both models, together with the differences in interpretation and applications.

Table S1: Specification of model 1 (with results given in the results’ section) and model 2 (alternative model with graphical results in Supplementary material S2)

|  | **Model 1** | **Model 2** |
| --- | --- | --- |
| 1^st^ level | $o_{it}\sim Poisson \left( \mu_{it}=e_{it}r_{it} \right);$  $log\mu_{it}=\log e_{it}+\log r_{it}$  $o_{it}=observed cases i-th LA$  $e_{it}=expected cases$ $i-th LA$  $r_{it}=relative risk to be modeled$ | $o_{it}\sim Poisson \left( \mu_{it}=e_{it}r_{it} \right);$  $log\mu_{it}=\log e_{it}+\log r_{it}$  $o_{it}=observed cases$i-th LA  $e_{it}=expected cases i-th LA$  $r_{it}=relative risk to be modeled$ |
| 2^nd^ level | $\log r_{it}= \beta+u_{i(j)}+v_{j}+\gamma_{t}+\delta_{jt}$  $\beta$ an overall risk level  $u_{i(j)}$spatial level i-th LA in j-th AC  $u_{i(j)}\sim N(0, \tau_{u})$ (exchangeable)  $v_{j}$ spatial level for j-th AC region  $v_{j} \sim N(0, \tau_{v})$ (exchangeable)  $\gamma_{t}$temporal effect t-th year  $\gamma_{t}$ random walk of order 1 RW(1)  $\delta_{jt}$space-time interact. with jth AC  $\delta_{jt}\sim N(0, \tau_{\delta})$ (exchangeable) | $\log r_{it}= \beta+u_{i}+v_{i}+\gamma_{t}+\delta_{it}$  $\beta$ an overall risk level  $u_{i}$ spatial level i-th LA  $u_{i(j)}\sim CARNormal$($0, \tau_{u})$^§^  $v_{i}$ spatial level for i-th LA  $v_{i} \sim N(0, \tau_{v})$ (exchangeable)  $\gamma_{t}$temporal effect t-th year  $\gamma_{t}$ random walk of order 1 RW(1)  $\delta_{it}$space-time interact. with jth LA  $\delta_{jt}\sim N(0, \tau_{\delta})$ (exchangeable) |
| 3^nd^ level | Precision parameters for all param  log(${}_{x}$) $\sim logGamma(1, 0.0005)$ | Precision parameters for all param  log(${}_{x}$) $\sim logGamma(1, 0.0005)$ |
| Interpretation of the model | Spatial correlation given by the nested structure (all HA within a region j share the effect $)$ | Spatial correlation given by neighborhood structure (the risk in i-th LA share information with the risk in neighbor LAs$)$ |
| Appropriate specially when | One of the main focus of the analysis is to assess the contribution of the region AC level and the differences of risk pattern evolution of each AC, where the political decisions are taken. | One of the main focuses is to know the spatial pattern, taking into account that neighbor areas may share similar underlying components such as risk factors or epidemiology characteristics. |

^§^ Besag J, York J, Mollié A. Bayesian image restoration, with two applications in spatial statistics. Annals of the Institute of Statistical Mathematics 1991, 43(1):1-20.

***Comparison of the characteristics of Model 1 (M1) and Model 2(M2)***

Table S2: Comparison of all components of the models

|  |  | **Component** | **Where to observe** | **Comparison** |
| --- | --- | --- | --- | --- |
| Spatially  correlated  structure | M1 | exp($v_{j}$) | Fig 1  upper row | Both show similar geographical pattern with marked regional effect. Model 2 more flexible, Model 1 more informative (regional governments have full responsibility on policy-making, planning and financing) |
|  | M2 | exp($u_{i}$) | Fig S1  upper row |  |
| Spatial (global)  structure | M1 | exp($v_{j}$+ $u_{i(j)}$) | Fig 1  Middle row | Practically identical results.  Marked geographical dependence |
|  | M2 | exp($v_{i}$+ $u_{i}$) | Fig S1  Middle row |  |
| Global time trend | M1 | ${exp(\gamma}_{t})$ | Fig 1 bottom row | Practically identical results. PCI and COPD exhibited opposite quasi-lineal trends, CCC an inverted-V shape |
|  | M2 | ${exp(\gamma}_{t})$ | Fig S1  bottom row |  |
| AC evolution  (at regional level) | M1 | $exp(v_{j}+\gamma_{t}+\delta_{jt}))$ | Fig 2 | Similar evolution patterns are shown per AC, with very few exceptions in regions with very few LA such as ‘lrj’, which in Model 2 are influenced by neighbor ACs. For M1, estimation of the AC effect and its trend is straightforward and comparable. For M2, estimation of the evolution at AC level is created a posteriori, as no such effect exists. |
|  | M2 | mean $\exp\left( u_{i}{+ v}_{i}+\gamma_{t}+\delta_{it} \right)$ per AC | Fig S2 |  |
| Global spatio-temporal evolution | M1 | $exp \left( {{\beta+ u}_{i\left( j \right)}+v}_{j}+\gamma_{t}+\delta_{jt} \right)$ | Fig 3-PCI  Fig 4-CCC  Fig 5-EPOC | The spatio-temporal pattern of the hospitalization risks reproduction is very similar in both models.  Agreement between the risk estimations by Intraclass Correlation Coefficient of agreement (ICC):  ICC - PCI: 0.90; CI_95_: (0.89, 0.91)  ICC - CCC: 0.94 CI_95_: (0.93, 0.94)  ICC- COPD: 0.95; CI_95_: (0.94, 0.95) |
|  | M2 | $exp \left( {{\beta+ u}_{i}+v}_{i}+\gamma_{t}+\delta_{it} \right)$ | Fig S3-PCI  Fig S4-CCC  FigS5-EPOC |  |

In conclusion, we show in this comparison study that the methodology presented here is flexible enough to answer specific research questions by adapting the specification of the model, and that the prediction of the global spatio-temporal patterns would not change depending on the specifications chosen. Depending on the hypothesized latent structures, more than one specification can give reliable results and can provide complementary information.

**Fig S1: Hospitalization risk maps of the spatially correlated component effect and of the global spatial component for Percutaneous Coronary Intervention (PCI; left), Colectomy in Colorectal Cancer (CCC; middle) and Chronic Obstructive Pulmonary Disease (COPD, right). At the bottom row, the average temporal trend (2002-2013)**


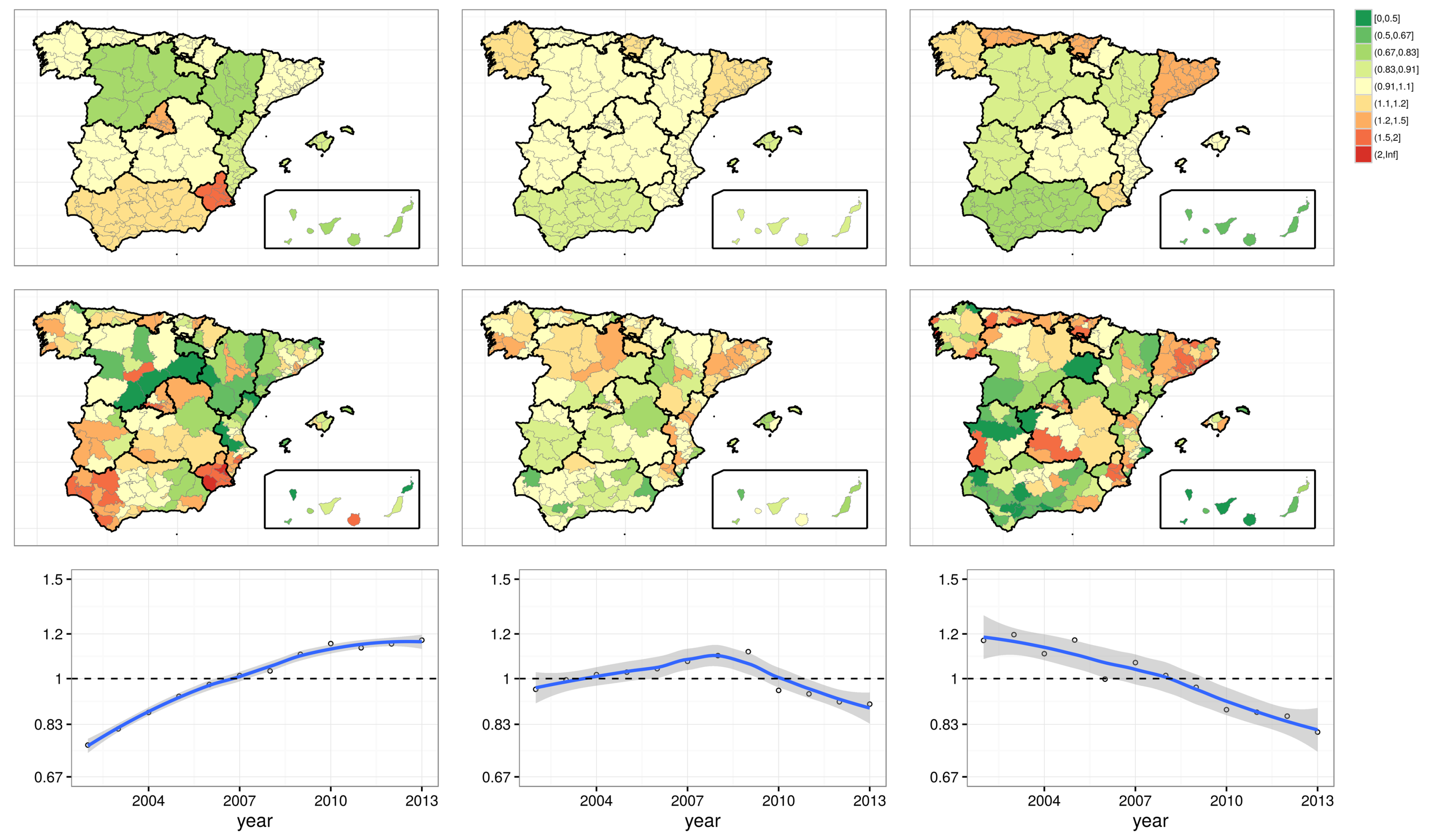

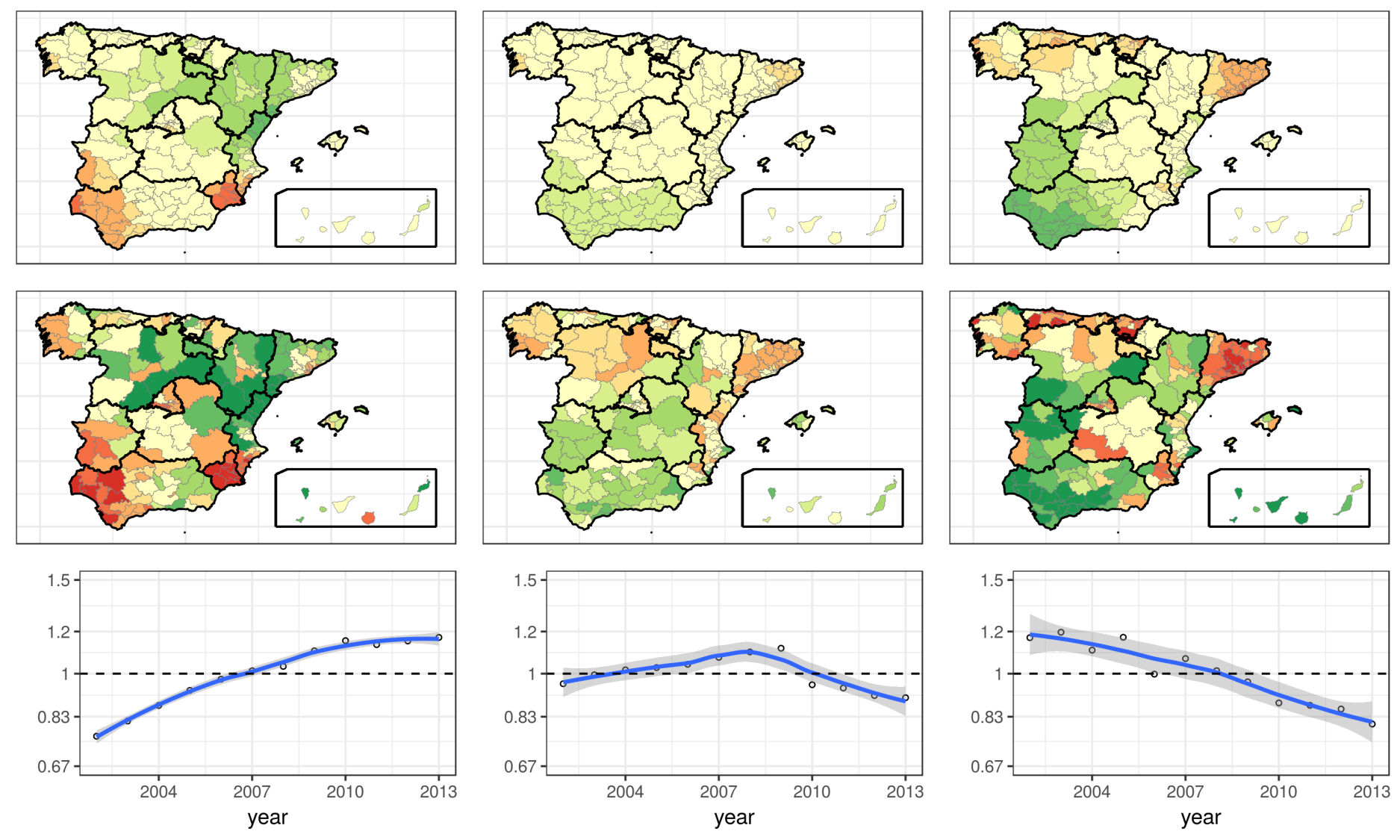


**Fig S2**: **Temporal trends for hospitalization risks in each of the 17 regions along 2002-2013 for Percutaneous Coronary Intervention (PCI; upper row), Colectomy in Colorectal Cancer (CCC; middle row) and Chronic Obstructive Pulmonary Disease (COPD; lower row)**


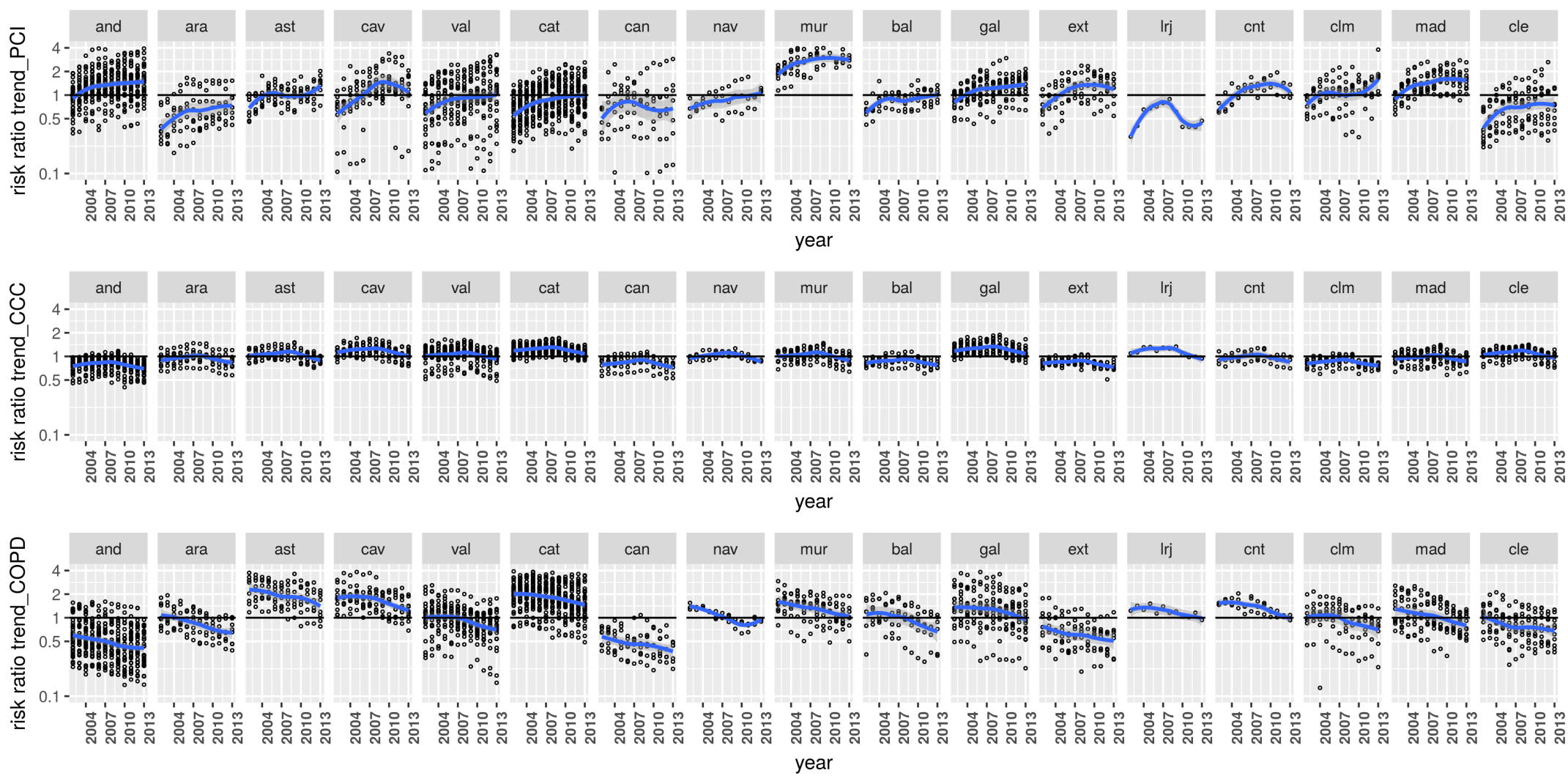


**Fig S3:** **Space-time relative risk estimates for Percutaneous Coronary Intervention (PCI)**

**Fig S4:** **Space-time relative risk estimates for Colectomy in Colorectal Cancer (CCC)**

**Fig S5: Chronic Obstructive Pulmonary Disease (COPD) admissions along 2002-2013**
